# North of England Medical Association

**Published:** 1840-01

**Authors:** 


### NORTH OF ENGLAND MEDICAL ASSOCIATION.

This is another of those Associations, now happily numerous in the land,* forced into existence by the profound conviction, almost universal among medical men, of the necessity of an important change in the general economy and government of our profession. We heartily congratulate our brethren on the accession of so numerous and influential a body to the great cause of medical reform, which, we doubt not, will, ere long, have many other similar aids to boast of. The North of England Medical Association was established at Newcastle on the 14th of November, at a meeting convened by a hundred of the most eminent medical men of the northern counties, and presided over by Dr. Headlam, of Newcastle The following resolutions show distinctly the nature and objects of the society:

"1. That, in the opinion of this meeting, the medical profession throughout Great Britain and Ireland has long been in a most unsatisfactory and anomalous condition; and that, owing to the defective nature of existing laws and institutions, consequences have ensued which imperatively demand the intervention of the legislature, being alike injurious to the profession, and incompatible with the welfare of society.

"2. That, with a view to submit to the notice of parliament the sentiments entertained by members of the profession at large, on subjects relating to their interests, and for the purpose of more effectually impressing upon the legislature the urgent necessity of reform in the medical institutions of this country, it is especially desirable that there should be union amongst medical practitioners.

"3. That, in order to adopt such measures as may facilitate the attainment of an improved system of medical government, as well as for the advancement of medical and surgical knowledge, and the promotion of friendly intercourse between practitioners residing in the northern counties, an association be now formed, under the title of the 'North of England Medical Association.'

"4. That a prominent feature in this Association shall be an active and zealous co-operation with other societies of a similar character throughout the United Kingdom."

* Two have been for some time established in Scotland—one at Glasgow, and one at Dundee. They are both highly respectable.

